# Implementing a cirrhosis order set in a tertiary healthcare system: a theory-informed formative evaluation

**DOI:** 10.1186/s12913-023-09632-z

**Published:** 2023-06-14

**Authors:** A. M. Hyde, E. Johnson, T. Luig, D. Schroeder, M. Carbonneau, D. Campbell-Scherer, P. Tandon

**Affiliations:** 1 Division of Gastroenterology (Liver Unit), Faculty of Medicine & Dentistry, University of Alberta, 8540 112 St NW, Edmonton, AB T6G 2P8 Canada; 2 Physician Learning Program, Faculty of Medicine & Dentistry, University of Alberta, Edmonton, Canada; 3 Alberta Health Services, Edmonton, Canada; 4 Department of Family Medicine, Faculty of Medicine & Dentistry, University of Alberta, Edmonton, Canada; 5 Office of Lifelong Learning and Physician Learning Program, Edmonton Clinic Health Academy (ECHA), 2-590, Edmonton, AB T6G 1C9 Canada

**Keywords:** Order set, Implementation, Formative evaluation, Implementation theory, Cirrhosis

## Abstract

**Background:**

Standardized order sets are a means of increasing adherence to clinical practice guidelines and improving the quality of patient care. Implementation of novel quality improvement initiatives like order sets can be challenging. Before the COVID-19 pandemic, we conducted a formative evaluation to understand healthcare providers’ perspectives on implementing clinical changes and the individual, collective and organizational contextual factors that might impact implementation at eight hospital sites in Alberta, Canada.

**Methods:**

We utilized concepts from the Consolidated Framework for Implementation Research (CFIR) and Normalisation Process Theory (NPT) to understand the context, past implementation experiences, and perceptions of the cirrhosis order set. Eight focus groups were held with healthcare professionals caring for patients with cirrhosis. Data were coded deductively using relevant constructs of NPT and CFIR. A total of 54 healthcare professionals, including physicians, nurses, nurse practitioners, social workers and pharmacists and a physiotherapist, participated in the focus groups.

**Results:**

Key findings revealed that participants recognized the value of the cirrhosis order set and its potential to improve the quality of care. Participants highlighted potential implementation challenges, including multiple competing quality improvement initiatives, feelings of burnout, lack of communication between healthcare provider groups, and a lack of dedicated resources to support implementation.

**Conclusions:**

Implementing a complex improvement initiative across clinician groups and acute care sites presents challenges. This work yielded insights into the significant influence of past implementation of similar interventions and highlighted the importance of communication between clinician groups and resources to support implementation. However, by using multiple theoretical lenses to illuminate *what* and *how* contextual and social processes will influence uptake, we can better anticipate challenges during the implementation process.

**Supplementary Information:**

The online version contains supplementary material available at 10.1186/s12913-023-09632-z.

## Background

Standardized order sets are based on clinical practice guidelines that guide healthcare providers in prescribing appropriate treatments and tests for patients admitted to the hospital [1]. Typically, standardized order sets offer direction for treating patients with conditions like cirrhosis, septic shock, or heart failure. These order sets affect the practices of physicians, nurses, and other healthcare providers throughout the patient’s hospital stay [2]. By using order sets, healthcare providers can improve adherence to guidelines, reduce in-hospital mortality, increase the quality of care, decrease variability in care practice, and reduce medication errors and length of stay [1, 3–10]. With the increased use of electronic medical records (EMRs), order sets embedded within these technologies have been identified as promising for improving patient outcomes and benefiting the healthcare team [3, 9]. While there are a few studies that have implemented a cirrhosis order set (see Table 1) at a single-center [11–13], there are no studies that have taken a broad approach, implementing a cirrhosis order set at multiple hospital sites within a large healthcare organization [2]. Further, we need to expand our lens to consider the pre-implementation conditions, particularly in the context of pragmatic trials intended to test the effectiveness of an intervention in a real-world clinical setting [14].

**Table 1:** Clinical case: cirrhosis & the CCAB trial

• Cirrhosis is a chronic condition resulting from vascular & hepatocellular injury.
• It leads to high rates of morbidity and mortality, and significant impairments to quality of life for patients [15, 16].
• Cirrhosis is commonly caused by factors like alcohol, Hepatitis C, and non-alcoholic fatty liver disease [3, 17].
• Patients with cirrhosis can experience complications like ascites, hepatic encephalopathy and variceal bleeding which are associated with high rates of acute care and resource utilization [18–23].
• The Cirrhosis Care Alberta (CCAB) pragmatic trial involves implementing a standardized order set across multiple acute care sites, supported by knowledge tools and resources for providers and patients, and aims to improve guideline-concordant care and clinical outcomes in Alberta [24].

As with any complex intervention in healthcare, implementation, scale, spread and sustaining change is challenging. Healthcare systems are emergent structures in complex adaptive systems [25, 26]. They are not systematically derived and cannot be conceived as complicated machines; rather, they are characterized by culture, communication networks, and local contextual realities which influence the daily interactions involved in the work required to provide high-quality patient care [27, 28]. Implementing innovations like order sets is entangled with contextual factors like organizational resources, culture, communication networks, organizational policies, leadership and physical environment [29, 30]. Further, these factors may be influenced by the social processes within an organization and individuals’ readiness and approach to change, all of which may fluctuate over time [31, 32]. There is a need to understand implementation context(s), including perceptions of the proposed intervention, social factors, capabilities and relationships of individuals, and organizational culture and capacity before the introduction of an intervention [33–35] to understand how context might impact the implementation process and identify strategies to overcome perceived challenges [36, 37].

We conducted a real-world formative evaluation of a complex intervention across multiple hospitals in an extensive health system. Formative evaluations not only present an opportunity for the research team to engage with stakeholders at individual study sites, but they also enable an understanding of how factors within complex contexts may potentially or actually influence the implementation of the intervention [38, 39]. Further, they can provide a clear linkage between pre-implementation context, implementation strategies and outcomes, emphasizing mechanisms of action and enhancing understanding of contextual factors impacting the implementation [40]. A thorough understanding of pre-implementation conditions is crucial to ensuring a systematic, measured approach to implementation and is also necessary to sustain and spread the intervention in other clinical settings [41, 42].

This study aimed to explore healthcare provider perspectives on (i) implementing standardized order sets, and (ii) the contextual and social factors at the individual, collective and organizational levels which will contribute to implementation effectiveness, with these insights used to inform our implementation strategy.

## Methods

A qualitative descriptive design [43] was adopted in this study. This study was part of a larger pragmatic trial (Clinical Trials Info) that received ethics approval from the University of Alberta Health Research Ethics Board (Pro00094054). Our results are reported according to the COREQ framework [44].

### Guiding theory & framework

This formative evaluation was guided by Normalization Process Theory (NPT) and the Consolidated Framework for Implementation Research (CFIR) to provide a nuanced understanding of both the contextual factors and social processes that affect implementation [31] (SEE Additional file 1). CFIR is a framework intended to comprehensively assess factors that may influence intervention implementation and effectiveness and has been used extensively in pre- and post-implementation evaluations [29, 45]. While CFIR comprises 39 constructs, divided into five domains, including (i) characteristics of the intervention, (ii) inner setting, (iii) outer setting, (iv) characteristics of individuals, and (v) implementation process [29], for this pre-implementation study, we focused on understanding clinician’s perspectives on the intervention and how their work setting may influence uptake. This corresponded to the domains of intervention characteristics, inner and outer settings, and characteristics of individuals. To expand upon the process components of CFIR, we used NPT. This theory focuses on the work of individuals and groups to implement an innovation, including how they incorporate novel interventions into their everyday work, what work is involved, and what structural supports and cognitive processes are required for implementation [32, 46]. For this pre-implementation study, the domains of coherence and cognitive participation in NPT were relevant, with the others (i.e., collective action and reflexive monitoring) helpful in understanding social processes after implementation.

### Participants

We purposively recruited healthcare professionals, including physicians, nurses, social workers, dieticians, and physiotherapists who provided care to patients hospitalized with cirrhosis across the eight hospitals in Alberta, Canada involved in the Cirrhosis Care Alberta (CCAB) project. Participants were recruited between October 2019 and February 2020. They were chosen for their clinical expertise and ability to provide insight into the current care for hospitalized patients with cirrhosis, including the context in which care is provided and their experiences implementing other similar quality improvement initiatives.

Potential participants were identified via project site leads who were asked to share an email invitation with any clinical or administrative staff with experience caring for patients admitted to the hospital with cirrhosis. Consideration was given to participant demographics to ensure a diverse group of healthcare professionals, including the type of professional (i.e., physician, nurse, etc.), length of time in professional role, and job type (ie, administrator, manager, front-line healthcare professional).

The research team was made up of seven researchers: AH, a Registered Nurse with expertise in qualitative research; EJ, a research assistant; TL an anthropologist with implementation and qualitative research expertise; DS, a Registered Nurse with expertise in qualitative and implementation science research, MC, a Nurse Practitioner with expertise in cirrhosis care, DCS, a primary care physician with expertise in qualitative and implementation science research, and PT, a hepatologist with expertise in cirrhosis and development of a clinical quality improvement initiative.

#### Data collection

We selected focus groups as our data collection method in recognition of their strengths in gathering diverse viewpoints and crucial group interaction data efficiently [47, 48]. One in-person focus group was conducted for each of the eight sites, with the groups lasting 30–120 min in length. Immediately before the focus groups, participants were provided with a 15-min overview of the CCAB by the principal study investigator (PT) that detailed the order set and other aspects of the intervention. At the outset of the focus groups, the focus group lead (AH) reviewed the study details and obtained informed consent from all participants. Sociodemographic information, including age, sex, professional role, and length of time in the professional role, was collected from participants.

Focus groups were guided by a semi-structured interview guide based on relevant NPT and CFIR constructs (see Additional file 1 for the interview guide). A research assistant (EJ) recorded detailed field notes, including contextual insights and group interactions [49]. Focus groups were recorded via digital recorder and transcribed verbatim.

#### Data analysis

Data collection and analysis were conducted concurrently to enable the refinement of our interview guide. We used a deductive content analysis approach in recognition of the explanatory nature of NPT and CFIR and their potential to produce actionable findings that could influence implementation [50]. First, our research team members (AH, EJ, DCS) developed our NPT and CFIR coding framework based on previous work by DS, TL, and DCS [51]. Transcripts were read and independently coded by AH and EJ, with team meetings held throughout the analysis process to review our framework and resolve discrepancies in coding. Lastly, we examined the coded data, including how it could be interpreted using NPT and CFIR [31]. Table 2 presents NPT and CFIR characteristics that were relevant to our analysis.

**Table 2:** Definitions of core constructs of CFIR & NPT identified in analysis

**Theory/Framework**	**Domain**	**Construct**	**Definition**
CFIR	Intervention characteristics:	Evidence strength & quality	Stakeholder perceptions of the evidence supporting an intervention
CFIR		Relative advantage	Participant’s perceptions of the intervention as being advantageous over current practices or an alternative solution
CFIR		Complexity	Relates to the perceived difficulty of implementing the intervention, including how long it might take, how work processes may be altered, who needs to be involved and potential disruptions to routine
CFIR:	Outer setting:	Patient needs & resources	The extent to which patient needs are prioritized by the organization, including the barriers and facilitators that may impact care
CFIR:		External policies & incentives	Those policies and/or incentives that are outside the organization and serve to spread or increase the uptake of an intervention
CFIR:	Inner setting:	Networks & communications	The nature and quality of communications within a site, specifically the quality of social networks between hierarchies or provider groups
CFIR		Implementation climate	Encompasses factors like the organization’s absorptive capacity for change, compatibility, relative priority for the implementation, and how use of the intervention will be rewarded within the organization
CFIR		Readiness for implementation	Includes the immediate indicators that an organization is committed to the decision to implement a particular intervention including leadership engagement, dedicated resources for implementation and access to information and knowledge to support implementation
CFIR		Available Resources	Resources dedicated to support implementation including money, training, education, and time
CFIR	Characteristics of Individuals	Individual knowledge and beliefs about the intervention	Attitudes & values toward the intervention and familiarity with the facts underlying the intervention
NPT		Coherence	The sense-making work of individuals & groups to understand the benefits of an innovation and what people need to do to use it
NPT		Cognitive Participation	The relational work that occurs to involve the right people in implementing an innovation

We used several strategies to enhance the rigor of our data collection and analysis processes [52].

A detailed audit trail and reflexive notes were maintained throughout the research process, and we involved several research team members in the collection and analysis phases to ensure agreement in coding and cross-coding (investigator triangulation) [53]. Data were managed using Quirkos [54].

## Results

Fifty-four healthcare professionals from 8 hospital sites participated in the focus groups, with focus groups ranging in size from 3–12 participants. All participants had experience providing care to patients admitted to the hospital with cirrhosis. Table 3 provides characteristics of study sites and demographic characteristics of participating healthcare providers.

**Table 3:** Site characteristics & demographic data

*Site*	Dedicated liver unit	Total (n)	Physicians/Nurse Practitioners (%)	Nurses (%)	Allied health professionals (Social workers, pharmacists) (%)
Hospital 1 (H1)	Yes	6		6 (100)	
Hospital 2 (H2)	No	3		3 (100)	
Hospital 3 (H3)	Yes	12	1 (8)	11 (92)	
Hospital 4 (H4)	No	8	4 (50)	4 (50)	
Hospital 5 (H5)	No	7	2 (23)	4 (57)	1 (14)
Hospital 6 (H6)	No	7	3 (43)	4 (57)	
Hospital 7 (H7)	No	6		5 (83)	1 (17)
Hospital 8 (H8)	Yes	5	1 (20)	3 (60)	1 (20)
		54	11 (20)	40 (74)	3 (6)

The perspectives of healthcare providers from across study sites, in conjunction with CFIR and NPT focus of our inquiry, resulted in three questions that structure our findings: (i) What is the cirrhosis order set, and why is it important? (ii) Who will be impacted, and how will it affect work? and (iii) How will the cirrhosis order set be supported? These questions and the supporting data acknowledge the complex interplay of contextual factors and social processes in implementing a novel innovation in the healthcare environment. Table 4 shows representative quotes of constructs from CFIR and NPT.

**Table 4:** Exemplar quotes from our findings

	**Theory or Framework**	**Relevant NPT or CFIR construct**	**Representative Quotes**
**What is the cirrhosis order set? Why is it important?**	CFIR	Evidence strength & Quality	“…when you have evidence born out of studies that show our outcomes are actually better and reducing outcomes like length of stay and admissions. The cerebral part of my brain says you should use them” (Physician H7)
	CFIR	Relative advantage	“I think order sets are a nice way to ensure things aren’t missed. People do things regularly and on a routine basis, even then things get missed. Having an order set with what you need to remember to put on an admissions or medication wise, I think that’s very helpful” (Physician, H8).
	CFIR	Individual knowledge & beliefs about the intervention	“Uptake varies. With the physicians as well as the residents, I feel like a lot of the residents don’t even know about the order sets. It does hinder the use of the order sets” (Manager, H5).
	CFIR	Intervention Complexity	“Typically, order sets are very long, like way too long. They are quite extensive and it takes a lot of time. You can’t expect front-line healthcare providers to complete that. There’s so much to it” (Manager, H3).
	CFIR	Patient Needs & Resources	“We do believe this is an important part of the population that we do see, but we don’t see them [patients with cirrhosis] in droves, right? I think part of our struggle, from an oversight view, is how we do everything [use order sets for other chronic conditions] and how do we do it well? (Manager, H1)
	NPT	Coherence	“Nurses love order sets, because we follow them and it’s nice to know that there’s nothing missed” (Nurse, H4). “I think they’re [order sets] great. Certainly, from a physician’s point of view, it’s very efficient” (Physician, H8)
**Who will be impacted & how will it affect work?**	NPT	Cognitive Participation	“There’s constantly changes in policy, charting or introducing new initiatives, it’s just so hard to keep up. If you’re adding new things [like a standardized order set], then something else will be pushed aside” (Nurse, H8)
	CFIR	Relative Advantage	“It simplifies things and makes it less likely that you’re going to miss something. It helps to standardize care, and it will make it easier for me because rather than needing to find the dosages of medications, it’s all right there”. (Physician, H6).
	CFIR	Implementation Climate	“There’s always the point of view that we’re implementing something new, and we get a lot of heel dragging and resistance because it’s new and people don’t want to add anything more to what they are doing. It’s a workload issue” (Manager, H6).
	CFIR	Networks & Communication	“Uptake varies. With the physicians as well as the residents, I feel like a lot of the residents don’t even know about the order sets. It does hinder the use of the order sets” (Manager, H5).
**How will the cirrhosis order set be supported?**	CFIR	External Policy & Incentives	“The uptake is poor with other order sets for COPD and HF. I found that our stroke order set has a different monitoring group that provides feedback and more rigorous stats. I think this helps with uptake” (Physician, H6).
	CFIR	Available Resources	“Staff feel like they’re doing more with less…There’s regular policy changes and new initiatives, it’s so hard to keep up. Without extra supports, something will end up falling off” (Manager, H3)
	CFIR	Implementation Climate	“So because of all the things that are currently happening and the pressures that we’re feeling related to healthcare funding, it’s going to be challenging. I don’t want it to become an onerous task for the staff, so I need to be realistic in how we implement this order set” (Manager, H7).
	CFIR	Readiness for Implementation	“I think the lack of staff is a huge piece. Now, I know that other initiatives have received extra staff, but I wonder if cirrhosis will receive that?” (Manager, H2)

### What is the cirrhosis order set? Why is it important?

Participants across all study sites were familiar with using standardized order sets to guide acute care management of chronic diseases as they had experienced the implementation of paper-based order sets within the last 10 years. They generally recognized the inherent value of order sets and their potential to make significant “*improvements in patient care*”. They associated the use of order sets with improved clinical outcomes like “*reduced length of stay and admissions,”* with data serving as a compelling reason for their use.

Others noted that order sets presented an opportunity for increased clinical efficiency and as a means of “*simplifying*” care. Further, they saw order sets as a means of improving patient safety. They shared:

*“…[order sets] simplify a lot of things, so it’s less likely that you’re going to miss something. If it’s handwritten, it can be misread…it just helps to standardise care”* (Pharmacist, H5).

Their past experiences framed participants’ understanding of the cirrhosis order set with similar innovations. Specifically, they were concerned with the “*oversaturation of order sets*” in a clinical environment crowded with order sets for stroke, diabetes, heart failure (HF), and chronic obstructive pulmonary disease (COPD). One participant shared:

*“...the order sets are very long, way too long...it takes a lot of time and you can’t expect front-line nurses to complete that”* (Nurse, H1).

Despite these concerns, most participants felt that implementing the cirrhosis order set made sense, with clear benefits evident to patients and providers. Several participants commented on the “*expansive and inclusive*” admission and discharge portions of the order set, noting that they would ensure “*holistic care*.” They valued the guidance contained within the order set on the “*dosing of drugs*” and “*ordering of lab tests*,” with one participant sharing: “*Nurses love order sets because we follow them and it’s nice to know that there’s nothing that’s missed*”.

Further, they recognized that a cirrhosis order set would help a vulnerable patient population that experienced frequent and prolonged hospitalizations. Though they acknowledged that patients with cirrhosis, were seen less frequently than other chronic disease populations like COPD and HF, they “…*believe that this is an important part of the population they see*”. One nurse reflected on this:

*“They’re [patients with cirrhosis] coming in with ascites and we’re draining a litre or 2 and they go back home and don’t change their diet...Then they’re coming back within weeks for the same thing”* (Nurse, H2).

They believed an order set, with guidance on a standardized approach to caring for patients with cirrhosis, was one means of improving the quality of care, and decreasing hospital readmissions.

### Who will be impacted and how will it affect work?

Improving the quality of care for patients with cirrhosis was identified as a priority for participants across all study sites. They recognized that they “*have a lot of cirrhotic patients*” and that implementing a cirrhosis order set was an important and necessary undertaking. The order set was perceived as an “*efficient*” means of enhancing care that would ensure care “*wasn’t being missed*”. Many participants were “*enthusiastic*” and “*very keen to learn*” about implementation of the order set, with one physician sharing:

*“I’m actually excited about this for all the reasons-you’ve got a clear order set. It seems to be really intuitive”.* (Physician, H3)

Others, however, acknowledged that the implementation of other order sets colored their attitudes toward the cirrhosis order set. They reported feeling “*burnt out*” and “*frustrated*” by the constant cycles of change. One participant reflected on this:

*“…there’s regular policy changes, and there’s new initiatives, you’re just changing constantly. It’s just hard for staff to keep up or something’s falling off*” (Nurse, H3)

Further, they shared concerns with challenges in communication between clinician groups and noted this as a barrier to uniform uptake of past order sets. One unit manager reflected on the challenges they encountered with communication with a past implementation:

*“Uptake varies especially with physicians and residents. A lot of the residents don’t even know about it [the order sets], so that does hinder their use…and if the interdisciplinary team is not involved in filling out their portion, then half of it is not completed…there’s a lot of gaps in terms of the entire team being actively engaged in the order sets” *(Unit Manager, H5).

Despite these concerns, participants believed they could integrate the cirrhosis order set into their daily practice and that it would become a “*routine*” part of care for patients with cirrhosis.

*“It sounds like it [the cirrhosis order set] is embedded in the EMR and you can print your discharge summary and patient education. As a nurse, the more things I have to find and remember, the much less chance that it happens, especially if the patient has multiple conditions. This [the cirrhosis order set] is much easier to do. The more that’s handed to you, the better”.* (Nurse, H8).

Healthcare providers also discussed the need for collaborative practice with a “*coordinated rollout*” across health disciplines, noting that focused implementation within sole provider groups resulted in confusion and disjointed patient care.

“*Sometimes the nurses get something and the physicians don’t know about it and that doesn’t go well. Or, the physicians are really excited about a tool and the nurses don’t know what it is, so it doesn’t work well either”* (Physician, H5).

Though all study sites were part of the same larger provincial health organization, it was evident that there were key differences in perceptions of interventions (like order sets) to improve the quality of care and contextual factors that might impact implementation. Participants recognized the potential impacts of order sets on their existing work processes. This, in combination with the patient load and complexity, influenced the overall willingness of the site to engage in such improvement initiatives. One participant shared:

*“I think we’ve not been the most cooperative site from the physician side of things…we’ve not been supportive of order sets…we’re saying we can’t fill out six different order sets…that is just killing us*” (Physician, H3).

This view was in contrast to other sites, which reported implementation of similar interventions having “*a concerted effort*”, with staff being “*very keen to learn*”.

### How will the cirrhosis order set be supported?

Despite the number of order sets and extensive implementation campaigns across all study sites, no policies or regulations mandating the use of order sets, with the onus for use placed on individual healthcare providers. Participants saw this as potentially hindering implementation, noting the importance of policy and institutional culture in the uptake of order sets. One participant reflected on institutional culture and ongoing monitoring of paper-based order set use:

*“The uptake [of order sets] is still poor within our physician group especially for the COPD and heart failure order sets. For our stroke order set, we have different monitoring with more rigorous stats, and the uptake has been greater” *(Physician, H4).

Participants also expressed worries about “*change fatigue*” and the overabundance of order sets and quality improvement initiatives. One nurse who worked on a general medicine unit that instituted order sets for congestive heart failure and chronic obstructive pulmonary disease worried about the impact of adding another order set:

*“I think part of our struggle, from an oversight view, is how do we do everything and how do we do it well, right. There are pressures related to the new government, I don’t want this [use of the order set] to become an onerous task for staff” *(Nurse, H7).

In reflecting on the cirrhosis order set and past order set implementation, participants highlighted the importance of having buy-in from organizational leadership and leadership from each healthcare provider group. Across the larger provincial health organization, the cirrhosis order set has been identified as a priority initiative for implementation, with upper leadership endorsing it and encouraging sites to incorporate it into their clinical care for patients with cirrhosis. Similarly, the implementation of this initiative was supported by medical leadership at all hospital sites, with participants expressing that there were gaps in care for patients with cirrhosis. Though most sites had strong front-line leadership support, leaders at several sites shared concerns that leadership was not unified amongst provider groups. That is, there was strong support for use of the cirrhosis order set amongst nursing leadership and front-line staff, but not always the same support from physician leadership. One nurse leader reflected on this:

*“Physician leadership really helps. You can tell who has good structure within their department because things flow, there’s communication, they [physicians] hear about it at their division meetings and they have their marching orders [to use it]” *(Nurse Manager, H1).

Resources, including education, time and personnel to support implementation, were identified as being crucial to the success of the cirrhosis order set. Participants felt that adding personnel and funding to support implementation would decrease “*frustration*” amongst staff and the perception of “*doing more with the same or what feels like less*”. Furthermore, they reflected on how aspects of the cirrhosis order set, like enhanced discharge education for patients, would be challenging for staff to implement given time constraints in an already busy clinical environment. One nurse shared:

*“It’s the capacity of the nurses and changing to a bit more proactive discharge planning and education. It was a struggle with the COPD pathway, and we’re continually working on it. So that [the cirrhosis order set] will bring some fresh problems [when it is introduced]” *(Nurse, H3).

## Discussion

Using CFIR and NPT, we gathered the insights of healthcare providers caring for patients with cirrhosis on their perceptions of our standardized order set and past implementation experiences. Our findings highlight the complexity of introducing a cirrhosis order set across a large provincial healthcare organization, illuminating potential implementation challenges related to organizational capacity and resources, involvement of different clinician groups and disruption of routine clinical care. Conducting a pre-implementation study allowed us to improve our initial implementation strategy [24] in a way that considers the intricate relationship between contextual factors and the implementation process while also being open to the unique needs of each hospital site.

A core focus of our formative evaluation was gathering clinician perspectives on the intervention itself: a standardized order set for hospitalized patients with cirrhosis. Though a standardized order set specific to caring for patients with cirrhosis is new in Alberta, participants reflected on their recent experiences with paper-based order sets for other chronic diseases like heart failure and COPD [55, 56] to frame their understanding of the cirrhosis order set. Despite the novel use of an EMR to deliver our intervention, healthcare providers still drew upon these past experiences to formulate their opinions on the cirrhosis order set. This tendency to draw upon past experiences was confirmed by previous studies highlighting the influence of ‘parallel’ initiatives and their potential contributions to implementation failure or a lack of engagement in the change process [57, 58]. Given the strong influence of these past initiatives, it is crucial to recognize the importance of the sensemaking work healthcare providers must do to understand the new order set and how it can be integrated into their current care practices for patients with cirrhosis.

Participants recognized that implementing the cirrhosis order set would require significant time and resources in an environment already plagued with capacity issues and “change fatigue.” They expressed concerns that though executive leadership identified the order set as a priority intervention across the larger healthcare organization, implementation would be challenging without additional resources like personnel and funding. Key to overcoming these contextual challenges will be a focus on fostering communities of engaged healthcare providers at each study site, identifying site champions [59], and creating awareness of the positive impacts of the order set [32, 60].

Increasingly, healthcare environments are being conceptualized as complex adaptive systems- dynamic networks of numerous human and non-human components interacting in a non-linear, somewhat unpredictable fashion [27, 28, 61]. This conceptualization acknowledges the inherent complexity of patients with chronic diseases like cirrhosis, the dynamic interactions between healthcare providers with differing priorities, and a healthcare system with internal and external pressures [62]. We illuminated the perceived complexities of implementing a cirrhosis order set across multiple hospital sites with different groups of healthcare providers; they recognized existing challenges in communication between physicians and nurses as a significant factor in past implementation of order sets. While healthcare providers endorse a shared goal: providing quality care to patients with cirrhosis, their approach to understanding and incorporating innovations, like standardized order sets, is not uniform. Therefore, our approach to implementation across clinician groups and study sites must be mindful of these communication networks (or lack thereof) and the social processes we can use to create a commitment to using the cirrhosis order set [31]. This complexity also precludes the identification of a singular implementation strategy that will be effective across all of our study sites. Instead of a one-size-fits-all approach, we need to work with our study sites to create tailored implementation plans that consider each site’s strengths and weaknesses and the diverse needs of the healthcare professionals.

This study builds upon the work of Schroeder et al. who proposed the integration of CFIR and NPT to better understand the influence of implementation process and contextual determinants on the uptake of an intervention within a complex adaptive system [31]. In our study, this integrated approach allowed us to look beyond how healthcare providers viewed the cirrhosis order set and their clinical contexts to understand what processes may be required to facilitate successful implementation. A review by Kirk et al. found that most studies using CFIR did so during or after the implementation process, with only 8% using the framework to guide pre-implementation study [45]. Further, most CFIR pre-implementation studies focused on systematically understanding the context and identifying barriers and facilitators that may be encountered [63–69]. While this approach can certainly be useful in understanding the contextual factors that may positively or negatively influence implementation, we suggest that this approach with a singular framework like CFIR minimizes the complexity in our healthcare systems. Further, it dichotomizes contextual determinants as either barriers or facilitators to implementation and assumes equal importance of all determinants. By using NPT, we gained insights into potentially important social and implementation processes to understand how they may impact the uptake of the cirrhosis order set across different hospital sites.

Currently, little research is available that evaluates the implementation of standardized order sets in the acute care setting; we could locate no literature addressing the pre-implementation contexts and social processes that may impact uptake. A recent review [1] found 14 studies that evaluated the impact of standardized order sets in acute care, but these studies only focused on clinical and cost-effectiveness outcomes such as length of stay, mortality, readmission rates and prescribing errors. They did not examine the implementation processes and experiences of healthcare providers. While these clinical and cost-effectiveness outcomes are important, they do not convey the story of implementation and the work that healthcare providers must do to routinize these practices into their daily work; processes are key to explaining the degree to which clinical outcomes are achieved. A comprehensive examination of the pre-and post-implementation processes and experiences of healthcare providers supports developing strategies to improve implementation and the quality of care for patients.

## Limitations

Despite efforts to recruit a robust and diverse group of healthcare providers at each study site, focus groups did not have any physician or allied health representation at several study sites. While this is generally representative of the staffing mix in acute care settings, the additional participation of physicians whose practice will be particularly influenced by this initiative may have yielded additional insights into challenges we may face in implementation. Further, due to scheduling challenges, we could only conduct one focus group per study site with a mixture of healthcare providers. Though there was the potential for dominant voices in these groups related to participant status within the healthcare system (i.e., physicians vs. allied health professionals), researchers ensured questions were directed at the non-dominant voices, which encouraged lively discussion and participation by all attendees [70, 71].

It should also be noted that this study was conducted before implementing the cirrhosis order set. Though participants were provided with an introduction and overview of the cirrhosis order set at the outset of each session, they had not actually used the order set in practice. Therefore, influencing contextual factors and social processes presented in this study were solely focused on the pre-implementation phase and different factors may manifest during implementation. Future work will compare anticipated barriers and facilitators to those reported post-implementation. This study was conducted before the COVID-19 pandemic in March 2020. Though informal communication (beyond the scope of this study) with healthcare providers at each site has confirmed that our findings remain relevant, before formally commencing implementation at each site, we must consider how our implementation strategies must be altered to best suit the current practice contexts. We would like to acknowledge that while we used CFIR and NPT as guides for our study, we did not delve into all their constructs. As a pre-implementation inquiry focused on gathering the perspectives of the front-line healthcare providers using the cirrhosis order set in their daily practice, exploring the CFIR domains of inner and outer settings with organizational leaders was beyond the scope of this study. The characteristics of individuals were not considered in-depth as an individual’s self-efficacy, stage and motivation to engage in change are not static but rather fluctuate over time in relation to context and other influencing social processes [29]. We limited our exploration of NPT to two constructs (coherence and cognitive participation), as the remaining constructs (collection action and reflective monitoring) help us understand the process only after implementation.

## Conclusion

This formative evaluation study is the first to use an integrated theoretical approach (NPT and CFIR) to explore healthcare providers’ perspectives before introducing a standardized order set across multiple acute care sites. This work yielded insights into the significant influence of past implementation of similar interventions and highlighted the importance of communication between clinician groups and resources to support implementation. Implementing a complex intervention that impacts practice across clinician groups and acute care sites presents many challenges. However, by using multiple theoretical lenses to illuminate *what* and *how* contextual and social processes will influence uptake, we can better anticipate challenges during the implementation process.

## Supplementary Information

**Additional file 1.** Interview Guide.

## Data Availability

The dataset used in during the current study is available from the corresponding author upon reasonable request.
