# Supplementary material for: Implementing a cirrhosis order set in a tertiary healthcare system: a theory-informed formative evaluation
Source: BMC Health Serv Res. 2023 Jun 14;23:636. doi: 10.1186/s12913-023-09632-z (PMC10266314; doi:10.1186/s12913-023-09632-z)
Supplement: Supplementary file 1 — Additional file 1. Interview Guide. [file 12913_2023_9632_MOESM1_ESM.docx]

Additional file 1: Interview Guide

1. Can you tell me about your previous experiences with implementing an order set (ie: heart failure and COPD)?
   1. What did you like about using an order set? Any positive impacts on your workflow?
   2. What didn’t work well for you? Why?
   3. What would have made implementation in these cases easier? What would’ve you needed to be more successful?
2. Let’s now talk about the cirrhosis order set we just heard about:
   1. What do you think about it? Is it worthwhile?
   2. What do you like about it?
   3. What do you not like about it?
   4. What is different in the order set from your current work? What is similar?
3. I’m now going to ask you to anticipate how this order set will impact your practice:
   1. How will this change your workday?
   2. How do you think this might change your relationships with colleagues?
   3. What might be some difficulties you might have in using the order set?
4. I want to talk about how you might know if this order set is impacting your practice:
   1. How might you know if the order set is working well for you and your patients?
   2. What kind of data would you need to know that the order set is working? At what intervals? In what format?
5. What kinds of things do you need from us (the CCAB) team to make the implementation of this order set successful?
   1. What kinds of education would you like? Around which parts?
      1. For example: physician education around the alcohol use disorder panel and medications, which modalities would work best for you?
      2. For example: nurse education for when you’re providing discharge education for a cirrhosis patient (how detailed would you like this education to be, how is it best delivered so that many nurses can see it?)
